# Revisiting the value of pre-hospital tracheal intubation: an all time systematic literature review extracting the Utstein airway core variables

**DOI:** 10.1186/cc9973

**Published:** 2011-01-18

**Authors:** Hans Morten Lossius, Stephen JM Sollid, Marius Rehn, David J Lockey

**Affiliations:** 1 Department of Research, The Norwegian Air Ambulance Foundation, Holterveien 24, PO Box 94, N-1441 Drøbak, Norway; 2 Institute of Surgical Science, Faculty of Medicine and Dentistry, University of Bergen, Harald Hårfagres gate 1, PO Box 7804, N-5020 Bergen, Norway; 3 Emergency Division, Oslo University Hospital Ullevål, Kirkeveien 166, PO Box 4956 Nydalen N-0424 Oslo, Norway; 4 Akershus University Hospital, Nordbyhagaveien 30, N-1478 Lørenskog, Norway; 5 University of Oslo, Faculty Division Oslo University Hospital, Kirkeveien 166, PO Box 1130 Blindern, N-0318 Oslo, Norway; 6 London Helicopter Emergency Service, Royal London Hospital, Whitechapel Road, London E1 1BB, UK; 7 Department of Anaesthesia, Frenchay Hospital, Winterbourne, South Gloucestershire BS16 1, UK

## Abstract

**Introduction:**

Although tracheal intubation (TI) in the pre-hospital setting is regularly carried out by emergency medical service (EMS) providers throughout the world, its value is widely debated. Heterogeneity in procedures, providers, patients, systems and stated outcomes, and inconsistency in data reporting make scientific reports difficult to interpret and compare, and the majority are of limited quality. To hunt down what is really known about the value of pre-hospital TI, we determined the rate of reported Utstein airway variables (28 core variables and 12 fixed-system variables) found in current scientific publications on pre-hospital TI.

**Methods:**

We performed an all time systematic search according to the PRISMA guidelines of Medline and EMBASE to identify original research pertaining to pre-hospital TI in adult patients.

**Results:**

From 1,076 identified records, 73 original papers were selected. Information was extracted according to an Utstein template for data reporting from in-the-field advanced airway management. Fifty-nine studies were from North American EMS systems. Of these, 46 (78%) described services in which non-physicians conducted TI. In 12 of the 13 non-North American EMS systems, physicians performed the pre-hospital TI. Overall, two were randomised controlled trials (RCTs), and 65 were observational studies. None of the studies presented the complete set of recommended Utstein airway variables. The median number of core variables reported was 10 (max 21, min 2, IQR 8-12), and the median number of fixed system variables was 5 (max 11, min 0, IQR 4-8). Among the most frequently reported variables were "patient category" and "service mission type", reported in 86% and 71% of the studies, respectively. Among the least-reported variables were "co-morbidity" and "type of available ventilator", both reported in 2% and 1% of the studies, respectively.

**Conclusions:**

Core data required for proper interpretation of results were frequently not recorded and reported in studies investigating TI in adults. This makes it difficult to compare scientific reports, assess their validity, and extrapolate to other EMS systems. Pre-hospital TI is a complex intervention, and terminology and study design must be improved to substantiate future evidence based clinical practice.

## Introduction

Tracheal intubation (TI) to secure the airway of severely ill or injured patients is a critical intervention regularly conducted by emergency medical service (EMS) providers throughout the world. This activity is based on the assumption that, in keeping with in-hospital practice, a compromised airway should be secured as early as possible to ensure adequate ventilation and oxygenation. However, because pre-hospital environmental and infrastructural factors can be challenging, intubation success rates are variable [1]. When TI is performed incorrectly, it can provoke adverse events and may worsen outcome in some patient groups [2-4]. Even when performed correctly, suboptimal ventilation following TI may increase the risk of fatal outcomes in certain patient subgroups [5-9].

The use of pre-hospital TI is widely debated [see Additional data file 1], but the majority of TI-related studies are thought to be of limited value [10-12]. The core question therefore remains unanswered: does TI in the pre-hospital setting fail or result in adverse events at rates that exceed the benefits of adequately performed TI?

Rapid sequence induction (RSI) and TI are regarded as the standard of care for airway management during in-hospital emergencies. It seems reasonable that this practice should be applied in the pre-hospital phase to prevent delay in good oxygenation and ventilation. However, because of available expertise and pre-hospital external factors, several alternatives to RSI and TI are practised. Environment, equipment, procedures, provider competence, practical skills, and drug protocols vary between emergency rooms and emergency medical service (EMS) systems [13], among EMS systems [14,15], and within EMS systems [16,17]. These variations have been reported to influence the frequency and quality of TI and, in all likelihood, patient outcome [1,18].

However, the heterogeneity of procedures, providers, patients, systems and monitored outcomes makes the published scientific reports difficult to interpret and compare, and inconsistency in the types of data reported exacerbates the problem. To improve reporting, an international expert panel published a consensus-based, Utstein-style template for the uniform reporting of data on pre-hospital advanced airway management [19]. The template defines inclusion criteria along with 28 core variables and 19 optional variables for documenting and reporting data. The 28 core variables are in three groups: "system variables", "patient variables", and "post-intervention variables" (Table 1). In addition, the template recommends that 12 fixed-system variables be reported (Table 2) to accurately describe the particular EMS system from which the data were collected.

**Table 1:** The 28 core variables for uniform reporting of data from advanced airway management in the field

Data variable name	Data variable categories or values	Definition of data variable
*System variables*		
Highest level of EMS provider on scene	1 = EMS non-P 2 = EMS-P 3 = Nurse 4 = Physician 5 = Unknown	Highest level of EMS provider on scene, excluding any non-EMS personnel (e.g., bystanders, family etc)
Airway device available on scene	1 = BMV 2 = Extraglottic device 3 = ETT 4 = Surgical airway 5 = None 6 = Unknown	Airway devices available on scene and provider on scene who knows how to use it
Drugs for airway management available on scene	1 = Sedatives 2 = NMBA 3 = Analgetics/opioids 4 = Local/topic anaesthetic 5 = None	Drugs used for airway management, available on scene and someone competent to administer
Main type of transportation	1 = Ground ambulance 2 = Helicopter ambulance 3 = Fixed-wing ambulance 4 = Private or public vehicle 5 = Walk-in 6 = Police 7 = Other 8 = Not transported 9 = Unknown	Main type of transportation vehicle (if multiple chose vehicle used for the majority of the transportation phase)
Response time	Minutes	Time from Emergency Medical Communication Centre operator initiates transmission of dispatch message to first resource/unit time of arrival on scene of first unit as reported by first unit
*Patient variables*		
Co-morbidity	1 = No (ASA-PS = 1) 2 = Yes (ASA-PS = 2-6) 3 = Unknown	ASA-PS definition 1 = A normal healthy patient 2 = A patient with mild systemic disease 3 = A patient with severe systemic disease 4 = A patient with severe systemic disease that is a constant threat to life 5 = A moribund patient who is not expected to survive without the operation 6 = A declared brain-dead patient whose organs are being removed for donor purposes
Age	Years or months	Years, if patient <2 years then months
Gender	1 = Female 2 = Male 3 = Unknown	Patients gender
Patient category	1 = Blunt trauma (incl burns) 2 = Penetrating trauma 3 = Non-trauma (including drowning and asphyxia) 4 = Unknown	Dominant reason for emergency treatment.
Indication for airway intervention	1 = Decreased level of consciousness 2 = Hypoxemia 3 = Ineffective ventilation 4 = Existing airway obstruction 5 = Impending airway obstruction 6 = Combative or uncooperative 7 = Relief of pain or distress 8 = Cardiopulmonary arrest 9 = Other, specify	Dominating indication for airway intervention
RR initial	Number/ Not recorded	First value recorded by EMS provider on scene
SBP initial	Number/ Not recorded	First value recorded by EMS provider on scene
HR initial	Number/ Not recorded	First value recorded by EMS provider on scene
GCS initial (m/v/e)	Motor 1-6 Verbal 1-5 Eyes 1-4 Not recorded	First value recorded by EMS provider on scene See also GCS definitions
SpO2 initial, state: with or without supplemental O2	Number/ Not recorded 1 = Without supplemental O2 2 = With supplemental O2 3 = Unknown if supplemental O2	First value recorded by EMS provider on scene
*Post-intervention variables*		
Post-intervention ventilation	1 = Spontaneous 2 = Controlled 3 = Mixed 4 = Unknown	How is patient ventilated following airway management? If both spontaneous and controlled choose mixed.
Post-intervention SBP	Number/ Not recorded	First value recorded by EMS provider after finalised airway management
Post-intervention SpO2	Number/ Not recorded	First value recorded by EMS provider after finalised airway management
Post-intervention EtCO2	Number/ Not recorded	First value recorded by EMS provider after finalised airway management
Post-intervention SBP on arrival	Number/ Not recorded	First value recorded by EMS provider after patient arrives at hospital
Post-intervention SpO2 on arrival	Number/ Not recorded	First value recorded by EMS provider after patient arrives at hospital
Post-intervention EtCO2 on arrival	Number/ Not recorded	First value recorded by EMS provider after patient arrives at hospital
Survival status	1 = Dead on scene or on arrival 2 = Alive on arival 3 = Unknown	Patient survival status: EMS treatment and on arrival hospital
Attempts at airway intervention	1 = One attempt 2 = Multiple attempts 3 = Earlier attempts 4 = Unknown	Number of attempts at securing the airway with extraglottic device or ETI. Earlier attempts describe the situation where another EMS personnel has attempted to secure the airway before the current.
Complications	1 = ETT misplaced in oesophagus 2 = ETT misplaced in right mainstem bronchus 3 = Teeth trauma 4 = Vomiting and/or aspiration 5 = Hypoxia 6 = Bradycardia 7 = Hypotension 8 = Other, define 9 = None recorded	Problems and mechanical complications recognized on scene and caused by airway management. Physiologic complications (5, 6 and 7) are regarded as such if they were not present before airway intervention and were recorded during or immediately after airway management. The following definitions are to be used: hypoxia: SpO2 <90% bradycardia: pulse rate <60 bpm hypotension: SBP <90
Drugs used to facilitate airway procedure	1 = Sedatives 2 = NMBA 3 = Analgetics/opioids 4 = Local/topic anaesthetic 5 = None	Drugs used to facilitate the airway intervention. Select all that apply.
Intubation success	1 = Success on first attempt 2 = Success after more than one attempt and one rescuer 3 = Success after more than one attempt and multiple rescuers 3 = Not successful	Successful intubation defined as tube verified in the trachea. An intubation attempt is defined as attempted laryngoscopy with the intent to intubate
Device used in successful airway management	1 = Bag Mask Ventilation 2 = SAD 3 = Oral TI 4 = Nasal TI 5 = Surgical airway 6 = None 7 = Unknown	Device used to manage successful airway or device in place when patient is delivered at hospital/ED

ASA-PS, American Society of Anesthesiologists physical status; bpm, beats per minute; BMV, bag mask ventilation; ED, emergency department; EMS, emergency medical service; EtCO2, end-tidal carbon dioxide; ETI, endotracheal intubation; ETT, endotracheal tube; GCS, Glasgow coma score; HR, heart rate; NMBA, neuromuscular blocking agent; P, paramedic; RR, respiratory rate; SAD, supraglottic airway device; SBP, systolic blood pressure; SpO2, saturation of peripheral oxygen, TI, tracheal intubation.

As identified by an international expert group [19].

**Table 2:** Fixed system variables for uniform reporting of data from advanced airway management in the field, identified by an international expert group

Data variable name	Data variable categories or values	Definition of data variable
Population	Number	Population count in the primary response area of the EMS
Area	Number	Area in square km or square miles of primary response area of the EMS
Rural, urban, split	1 = Urban 2 = Rural 3 = Split	Urban area defined as: "De facto population living in areas classified as urban according to the criteria used by each area or country. Data refer to 1 July of the year indicated and are presented in thousands" Rural area defined as: "De facto population living in areas classified as rural. Data refer to 1 July of the year indicated and are presented in thousands"
Usual tiered response	Free text	Describe briefly
Time intervals collected	Free text	Describe briefly
Mission type	Free text	Describe briefly; e.g. Mainly trauma or mixed patient population
Times available	Free text	Describe briefly
Established airway management protocols	Free text	Describe briefly
Airway management techniques available	Free text	Describe briefly
Describe type of training in airway management	Describe briefly	
Type of tracheal tube confirmation technique	1 = Auscultation 2 = Colorimetry 3 = Capnometry 4 = Capnography 5 = None	
Type of available ventilator	Free text	Describe briefly

EMS, emergency medical service.

The aim of this study was to determine the rate of reported Utstein airway variables (28 core variables and 12 fixed-system variables) found in current scientific publications on pre-hospital TI [19].

## Materials and methods

### Study eligibility criteria

We included original English language articles pertaining to pre-hospital TI in adult patients. Studies that investigated pediatric cohorts and studies that focused on surgical airways were excluded. Studies that compared TI to other airway devices were also excluded.

### Identification and selection of studies: data extraction

A systematic search of Medline and EMBASE databases according to the PRISMA guidelines to identify all relevant studies published prior to 1 September, 2009 was conducted (see Table 3 for search strategy) [20]. All records were converted into an EndNote bibliographic database (EndNote X1^© ^Thompson Reuters, UK). Two reviewers (HML and MR) examined the titles and abstracts of the records for eligibility. The full texts of all potentially relevant studies were obtained, and two reviewers (HML and MR) assessed whether each study met the eligibility criteria. The reference lists of the included studies and a recent relevant Cochrane review were inspected to identify additional relevant studies [11].

**Table 3:** Search strategy for identification of relevant studies in Medline and EMBASE

Database	Search terms
	**"keywords"**
Medline	"emergency medical services" AND "intubation, intratracheal"
EMBASE	"emergency care" AND "intubation/or respiratory tract intubation"
	**"title"**
Medline	"prehospital" AND "intubation"
Medline	"pre-hospital" AND "intubation"
Medline	"out-of-hospital" AND "intubation"
Medline	"prehospital" AND "RSI" OR "rapid sequence induction"
Medline	"pre-hospital" AND "RSI" OR "rapid sequence induction"
Medline	"out-of-hospital" AND "RSI" OR "rapid sequence induction"
EMBASE	"prehospital" AND "intubation"
EMBASE	"pre-hospital" AND "intubation"
EMBASE	"out-of-hospital" AND "intubation"
EMBASE	"prehospital" AND "RSI" OR "rapid sequence induction"
EMBASE	"pre-hospital" AND "RSI" OR "rapid sequence induction"
EMBASE	"out-of-hospital" AND "RSI" OR "rapid sequence induction"

### Study characteristics

One reviewer (HML) used a standardised Excel spreadsheet (^© ^2007 Microsoft Corporation, USA) and extracted information from the included studies according to the newly published template for uniform reporting of data regarding advanced airway management in the field [19]. Reported variables that matched the Utstein variables were regarded as identical, although definitions sometimes differed or remained unreported.

The data were analysed using the Statistical Package for the Social Sciences, v. 18.0 (SPSS, Inc., Chicago, IL, USA), and the distributions were reported as medians and inter-quartile ranges (IQR). Being a systematic literature review, this study did not need approval from The Regional Committee for Research Ethics or the National Social Science Services.

## Results

### Literature search

**Figure 1:**
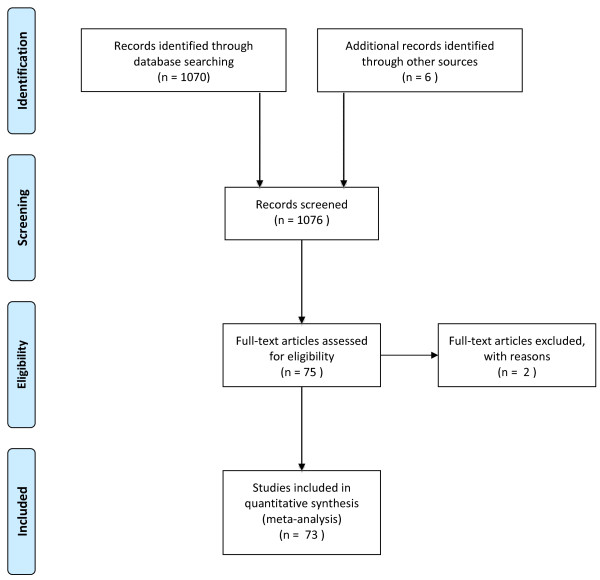
**A search diagram according to the PRISMA statement**.

We identified 1,070 records in the initial search. Another six records were identified through other sources. Among these 1,076 records, 75 full-text original papers were assessed. Two of these were excluded from further analysis, one because of qualitative methodology and one being a preliminary report, leaving 73 studies for the final analysis (Figure 1).

### Characteristics of the included studies

The majority of the studies (59, 81%) were from North American EMS systems. Of these, 46 (78%) described services in which non-physicians conducted TI. In contrast, 13 (87%) of the 15 non-North American EMS systems, physicians performed the pre-hospital TI. Of the 47 non-physician-manned systems, 25 (53%) performed drug-assisted TI.

Sixty-five studies had applied an observational methodology (89%), of which 29 were conducted prospectively and 36 retrospectively [see Additional data file 1]. We identified two randomised controlled trials (RCT) and six non-RCT interventional studies.

### Core variables

None of the included studies presented the complete set of 28 variables recommended in the template [19]. The maximum number of core variables reported in a single study was 21. The minimum number reported was two, whereas the median number of core variables reported from all the studies was 10 (IQR 8 to 12).

The most frequent reported core variable was "patient category", reported in 63 (86%) of the 73 studies (Table 4). The least reported variable was "co-morbidity", reported in only 2 (3%) of 73 studies (Table 4).

**Table 4:** Number of times (%) each Utstein variable was collected and documented among the 73 studies included

Core variables	Number (%)
*Core system variables*	
Main type of transportation	55 (75%)
Highest level of EMS provider on scene	34 (47%)
Airway device available on scene	26 (36%)
Drugs for airway management available on scene	27 (37%)
Response time	10 (14%)
*Core patient variables*	
Patient category	63 (86%)
Age	59 (81%)
Gender	53 (73%)
GCS initial (m/v/e)	40 (55%)
Systolic blood pressure, initial	35 (48%)
Indication for airway intervention	26 (36%)
Heart rate, initial	13 (18%)
Respiratory rate, initial	12 (16%)
SpO2 initial, state: with or without supplemental O2	11 (15%)
Co-morbidity	2 (3%)
*Post intervention variables*	
Intubation success	44 (60%)
Device used in successful airway management	41 (56%)
Survival status	40 (55%)
Complications	30 (41%)
Drugs used to facilitate airway procedure	28 (38%)
Attempts at airway intervention	25 (34%)
Post-intervention SBP on arrival	11 (15%)
Post-intervention SpO2 on arrival	10 (14%)
Post-intervention EtCO2 on arrival	8 (11%)
Post-intervention SBP	8 (11%)
Post-intervention SpO2	8 (11%)
Post-intervention ventilation	3 (4%)
Post-intervention EtCO2	3 (4%)
**Fixed system variables**	
Service mission types	52 (71%)
Established airway management protocols	48 (66%)
Area	40 (55%)
Usual tiered response	33 (45%)
Type of tracheal tube confirmation technique	31 (42%)
Rural, urban, split	31 (42%)
Airway management techniques available	30 (41%)
Population	24 (33%)
Describe type of training in airway management	23 (32%)
Time intervals collected	15 (21%)
Times available	13 (18%)
Type of available ventilator	1 (1%)

EMS, emergency medical service; EtCO2, end-tidal carbon dioxide; GCS, Glasgow coma score, SBP, systolic blood pressure; SpO2, saturation of peripheral oxygen.

### Fixed-system variables

Of the 12 fixed-system variables, the maximum number reported in a single study was 11. The median number reported was five (IQR four to eight), and two studies did not report any of the recommended fixed-system variables. The most frequently reported variable was "service mission type", which was reported in 52 (71%) of the 73 studies (Table 4). The least frequently reported fixed system variable was "type of available ventilator", which was only reported in one paper (1%) (Table 4).

All the studies included in the review are listed, and the number of matching core variables and fixed-system variables from each study are presented in Additional file 1.

## Discussion

Our systematic literature review of studies pertaining to TI of adults revealed deficient reporting of the Utstein airway core variables as defined by an international expert group. Recommended core variables, such as "post-interventional end-tidal carbon dioxide (ETCO_2_)", "number of attempts at airway intervention" and "co-morbidity", which are all recognised as being highly associated with efficiency and outcome, were missing in the majority of the papers. Fixed-system variables were incompletely reported or absent in most of the included studies. The low number of reported core variables makes it difficult to compare different scientific reports, assess their validity, and extrapolate to other EMS systems. One could claim that several of the included studies with a low number of documented and reported core variables in fact only report the occurrence and performance of TI within their system and therefore are not reflective of the effects or efficiency of pre-hospital TI.

**Figure 2:**
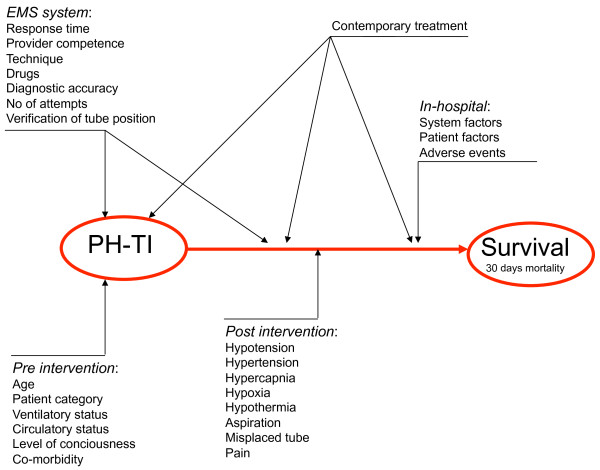
**A cause-effect chart and factors influencing the relation between PH-TI and survival**.

Several studies have focused on the intricacy of implementing TI in the pre-hospital setting [21-23]. TI represents a complex intervention (Figure 2) that contains several separate but highly interacting components. Scientific studies on this subject are difficult to design and interpret because of tremendous variability in (and insufficient description of) operator experience, technique, and patient case-mix, making it difficult to understand or eliminate confounding factors [24]. Furthermore, neither contemporary interventions nor pre-intervention, per-intervention, or post-intervention factors highly likely to influence outcome are usually documented, analysed, or adjusted for. Key in-hospital factors (likely to be concealed from the investigator) further confound the outcome analysis [25]. This finding may explain why apparently similar studies present conflicting results and reach opposite conclusions.

RSI with oral intubation is the standard of care for drug-assisted emergency TI because it is widely recommended to be the safest way of performing this high-risk intervention [26-28]. However, only 19 (31%) of the 73 papers in this study reported the variable "drugs for airway management available on scene". Among papers that reported this variable, the definition and extent of drug assistance varied. Some services had protocols based on administering a muscle relaxant only; some combined this with a small dose of a sedative or analgesic, whereas some administered a traditional RSI. The presence or absence of drug assistance and the availability and dose of specific agents are likely to influence the success rate of TI and the rate and severity of adverse events. This information is essential to correctly assess the reported outcomes.

The majority of the included papers were based on observational studies, commonly referred to as low-quality evidence [29]. In a complex intervention, a true association between a single cause (TI) and an effect (survival) is difficult to prove (Figure 2). The presented results are flawed by multiple confounding factors, and external validity is questionable. Even randomization may fail to exclude the major confounders, a phenomenon demonstrated by Gausche et al. in one of the few randomized trials on pre-hospital TI [30]. The investigators reported no additional effect on survival or neurological outcome when paramedics performed pre-hospital TI compared with traditional bag/valve/mask ventilation in critically ill pediatric patients. The study set out to analyze the effect of the intervention itself, but due to an "intention-to-treat protocol", the intervention group was heavily confounded (abstained intubation, repetitive attempts of intubation, or failed intubation). The study instead demonstrates the effects of suboptimal provider competence and TI complications, and it illustrates the challenges of using traditional analytical techniques when assessing a complex intervention.

Several recent reviews have assessed the evidence of a pre-hospital TI effect [10,31], including a Cochrane review [11]. They consistently conclude that the available evidence is limited and weak. It has been suggested that the traditional method of systematic review is of limited use in the evaluation of a complex intervention [32]. The lack of a standard definition of pre-hospital TI poses a significant challenge for systematic reviewers and readers of these reviews. With respect to the Cochrane review on pre-hospital TI [11], the number of studies located in our review illustrates that any strict inclusion criteria for a systematic review will exclude the majority of studies published because pre-hospital TI is often performed differently or described inadequately. It also questions the whole evidence base on which current practice is based.

### Limitations

We have assessed the included studies assuming that all the recommended Utstein airway core variables are important to document for each study. Some studies focus on particular aspects of pre-hospital TI intervention and may not need to report all the core variables from the template. Nonetheless, understanding the correlations between the intervention and its outcomes presupposes that all the interacting factors are accounted for.

The Utstein airway template still requires validation. Not all the variables relevant to outcome may have been identified. In a systematic review of studies on out-of-hospital cardiac arrest, a large variability in outcome not entirely explained by variability in documented Utstein variables, was found [33].

We also acknowledge that some relevant studies may not have been located during our database search. In the future, more homogenous reporting of studies pertaining to pre-hospital TI may reduce these limitations.

## Conclusions

Our systematic literature review of studies investigating TI in adults demonstrated that core data required for proper interpretation of results were frequently not recorded and reported. The inconsistent and imprecise reporting of data may be the explanation for the fact that, despite numerous published studies on this subject, there is an ongoing debate on if, when, how, and by whom pre-hospital advanced airway management should be performed. Pre-hospital TI is a complex intervention, and terminology and study design must be improved to substantiate future evidence-based clinical practice. To support this, there is a significant need for an international standard for documenting and reporting pre-hospital TI in severely ill and injured patients. The newly published template might be a first and important step in this direction [19].

## Key messages

• Studies investigating pre-hospital TI in adults lack the core data required for useful interpretation of results.

• The published studies investigating pre-hospital TI rarely present high-quality scientific evidence.

• Pre-hospital TI is a complex intervention, and terminology and study design must be developed to substantiate future evidence-based clinical practice.

• A recently published template for reporting advanced pre-hospital airway management might be a first and important step in this direction.

## Abbreviations

EMS: emergency medical services; ETCO_2_: end-tidal carbon dioxide; IQR: interquartile range; RCT: randomized controlled trial; RSI: rapid sequence intubation; TI: tracheal intubation.

## Competing interests

The authors declare that they have no competing interests.

## Authors' contributions

HML, SJMS and DJL developed the protocol. MR and HML conducted the systematic review. HML performed the analysis and drafted the manuscript. All authors read and approved the final manuscript.

## Supplementary Material

Additional file 1 **Overview of included studies**. Aim of study, study design, TI provider, continent, number of the 28 Utstein core and 12 Utstein fixed system variables (%) reported in the 73 reviewed studies. Click here for file
